# Egbert Guernsey, M. D., and Ward’s Island Hospital

**Published:** 1890-08

**Authors:** A. McNeil

**Affiliations:** San Francisco, California


### EGBERT GUERNSEY, M. D., AND WARD'S ISLAND HOMŒOPATHIC HOSPITAL.

A. McNeil, M. D., San Francisco, California.

In reading Dr. Guernsey's attempt to carry the hospital which it was his sacred duty to care for into the camp of the allopaths, it struck me to endeavor to find its parallel in American history. All know who have read history with what devotion soldiers will dare and suffer to defend the flag or other trust committed to their hands. All of us who have worn the blue can recall the feeling akin to worship with which we gazed on the old flag, in those days that tried men's souls, and to-day we feel our eyes moisten as on patriotic occasions we see it.

And in the annals of our country I recall two names only of men who were willing to hand over to the enemy the men and positions that have been confided to their hands, Benedict Arnold and General Twiggs. Was not Ward's Island Homœopathic Hospital committed to his fostering care because he was believed to be a homœopath? And when he ceased to be a homœopath, if he ever was one, that sacred trust was violated by the design on his part of carrying it over to the enemy. What is the difference between Guernsey on one hand and Arnold and Twiggs on the other? I confess I cannot see it with the naked eye.

